# Revealing GRK5 Activation Features by Interpretable Machine Learning and Molecular Dynamics Simulation

**DOI:** 10.3390/ijms27073329

**Published:** 2026-04-07

**Authors:** Yuanpeng Song, Ming Kong, Fuhui Zhang, Xuemei Pu

**Affiliations:** 1 College of Chemistry, Sichuan University, Chengdu 610064, China; 2023222030041@stu.scu.edu.cn (Y.S.); 2024222030028@stu.scu.edu.cn (M.K.); 2 Graduate School, Sichuan University, Chengdu 610064, China

**Keywords:** G protein-coupled receptor kinase, molecular dynamics simulation, machine learning, activation

## Abstract

G protein-coupled receptor kinase 5 (GRK5) is an important therapeutic target involving cardiovascular diseases, cancer, and inflammatory disorders. However, the features of its activation as an essential function regulation process have been poorly understood, limiting related drug development. The work utilizes a molecular dynamics simulation coupled with an interpretable machine learning model to identify key structure and dynamics determinants distinguishing the active and inactive states of GRK5. Benefiting from the unbiased and data-driven framework, the work reveals that the active site tether (AST) is a dominant activation-associated feature, acting as a conformational switch that regulates kinase domain movements. Beyond this canonical element, we also uncover two previously underappreciated structure modules contributing to GRK5 activation, such as the coupling interaction between the α10/α11 helix interface with the N-terminal lipid-binding domain (NLBD) in the active state, and the α5 helix region that facilitates large-scale RH domain reorientation. Conformation dynamics analyses further indicate that GRK5 activation involves disruption of the interdomain interactions and interaction coupling between AST, αN-helix, kinase domain N-lobe, NLBD, and α10/α11 hinge. These observations provide valuable insights into understanding the GPK5 activation mechanism and also highlight the power of machine learning in capturing functionally conformational changes, and in turn offering a methodological guideline for the studying of the protein function mechanism.

## 1. Introduction

G protein-coupled receptor kinases (GRKs) play essential roles in regulating G protein-coupled receptor (GPCR) signaling and a wide range of intracellular physiological processes. Based on sequence homology and functional characteristics, the GRK family is classified into three subfamilies: GRK1 subfamily (GRK1 and GRK7), GRK2 subfamily (GRK2 and GRK3), and GRK4 subfamily (GRK4, GRK5, and GRK6) [1]. Within the GRK4 subfamily, GRK5 is of particular biological interest owing to its broad tissue distribution, a unique nuclear localization signal (NLS), and its ability to regulate diverse signaling pathways through the phosphorylation of non-GPCR substrates [2,3].

Dysregulation of GRK5 activity has been implicated in the pathogenesis of multiple human diseases, including cardiovascular disorders, cancer, osteoarthritis, and neuropsychiatric conditions. For instance, modulation of GRK5 activity has been demonstrated to attenuate cardiac fibrosis [3,4,5], influence chemosensitivity in cancer cells [6,7,8], slow the progression of osteoarthritis [9], and regulate signaling pathways associated with neuropsychiatric diseases [10,11]. These studies highlight the functional importance of GRK5 activity regulation and have motivated continued interest in pharmacological strategies targeting GRK5 [12,13,14,15].

However, the activation features distinguishing the active state from the inactive one remain very limited, resulting in a poor understanding of the molecular mechanism underlying the activity regulation. Recent advances in structural biology, particularly through X-ray crystallography and cryo-EM, provide high-resolution structures of GRKs in multiple conformational states. These structures include canonical inactive conformations (e.g., GRK1–ATP, GRK2–CCG258747) [16,17] as well as receptor- or regulator-bound active states (e.g., GRK5–CaM, GRK1–rhodopsin, GRK2–NTSR1) [18,19,20]. Comparisons of these structures provide some important structure differences between the inactive and active states, for example, the ordering of the αN-helix and rearrangements of the active site tether (AST). However, the GRK activation is inherently dynamic, which is hardly obtained from these static structures. Therefore, capturing and characterizing these dynamic features are essential for understanding GRK5 activation.

Molecular dynamics (MD) simulation provides a powerful computational tool for exploring protein structure and dynamic changes at the atomic resolution [21,22,23,24], including GRKs [25]. However, existing MD studies on the GRK family have primarily concentrated on ligand screening or binding-related mechanisms of GRK2 and GRK5 [26,27] while their activation mechanisms have been severely under-investigated. In 2017, Yao et al. combined principal component analysis with multiple 100 ns MD simulations to explore the conformation landscape of GRK5 activation based on point mutations and residue truncation [28], yet the short simulation timescales and the use of artificial perturbations limit characterization of the realistic activation dynamics of GRK5. In fact, the activation of GRK is a long-timescale process that is generally inaccessible to conventional MD simulations. However, we can adopt a viable alternative that separately characterizes the conformation space of the active and inactive states. Then, we identify the key structural and dynamics determinants that distinguish the two conformation states, which can provide valuable insights into the underlying activation mechanism.

Motivated by the issue, we start from experimentally determined structures of inactive and active GRK5 and utilize MD simulation to obtain their respective conformational spaces. However, it is a technical challenge to unbiasedly identify the structural features distinguishing the inactive and active states from the high-dimension conformation spaces. To address the difficulty, we adopt an interpretable machine learning model to capture the differences. Finally, by combining principal component analysis (PCA) and Dynamic Cross-Correlation Matrix (DCCM) analysis, we further reveal critical dynamic signatures and residue-level contributions underlying the GRK5 activation.

## 2. Results and Discussion

### 2.1. Conformational Characteristics of Inactive and Active GRK5

To validate that our simulations reliably capture the key conformational features of inactive- and active-state GRK5, and to establish a foundation for understanding the underlying mechanistic dynamics, we built simulation systems based on the crystal structures of the inactive state (GRK5–sangivamycin, PDB: 4TNB) and the active state (GRK5–CaM, PDB: 6PJX) [18,29]. For each state, we performed three parallel 300 ns MD simulations to obtain the active and inactive conformation spaces so that we can capture their dynamic features. The three simulations exhibited similar change trends. Thus, in the text, we only select one representative trajectory in the data presentation involving structure change with simulation time, while results from the other two parallel trajectories are provided in the Supporting Information. Only for statistical analyses not needing presenting time evolution, did we use the concatenated trajectories from all three parallel simulations.

**Figure 1:**
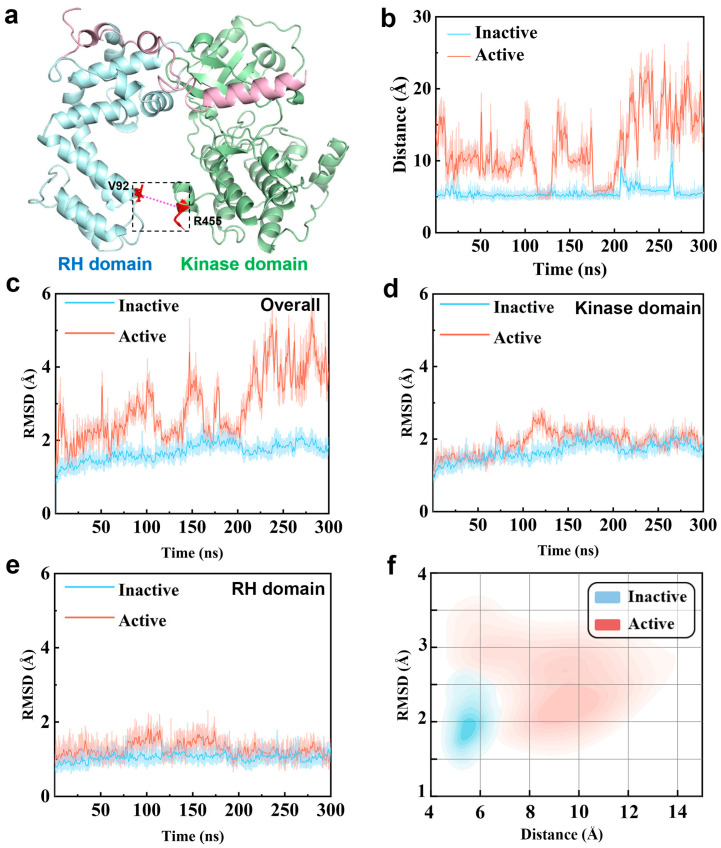
Comparison of structural characteristics between the inactive and active conformational ensembles of GRK5. (**a**) The GRK5 structure. (**b**) The time evolution of interdomain distance between the RH and kinase domain, characterized by the Cα–Cα distance between Val92 and Arg455. Blue represents the inactive state and red denotes the active state. (**c**) Time evolution of the backbone root-mean-square deviation (RMSD) for the overall structure. The inactive and active states are colored blue and red, respectively. (**d**) Time evolution of the backbone RMSD for the kinase domain. (**e**) Time evolution of the backbone RMSD for the RH domain. (**f**) A clustering projection of the concatenated three parallel trajectories for the active and inactive conformations, based on the RMSD values of the overall structure and the closest non-hydrogen atomic distance between Val92 andArg455.

As illustrated by Figure 1a, GRK5 mainly consists of two structured domains connected by flexible linker regions, such as a regulator of G Protein Signaling Homology (RH) domain and a kinase domain. The relative orientation and dynamic coupling of these two domains were considered important factors in regulating activation [30,31]. Structural studies already identified some interactions at the interface between the RH and kinase domain, sometimes referred to as “ionic lock”, which is taken as an important structural feature maintaining the inactive state of GRK5 [30]. Disruption of the interaction would lead to domain separation, marking the active state [18,28]. Thus, we use the Cα–Cα distance between Val92 in the RH domain and Arg455 in the kinase domain (vide Figure 1a) as a structural metric to characterize the interdomain change during the simulations, as shown by Figure 1b and Figure S1.

As reflected by Figure 1b, in the inactive state simulations, the Val92–Arg455 distance is primarily maintained at ~5 Å. Although transient fluctuations occur, they do not exceed 10 Å, indicating that interdomain interactions remain stable within the simulation time. In contrast, the active state simulations show a markedly different pattern: the distance and its fluctuation amplitude increase significantly, frequently exceeding 20 Å and reaching a maximum of ~30 Å. The observation is in line with the disruption of the interdomain interactions observed in experimental active state structures [18,32], indicating that the conformation ensemble of the active state derived from our simulation can reasonably reflect its core structural features.

To evaluate global conformational differences, we also calculate the backbone root-mean-square deviation (RMSD) relative to their respective initial structures (Figure 1c and Figure S1). The active state exhibits significantly larger RMSD fluctuations than the inactive state, in line with the difference in the interdomain distance between the active and inactive states. To distinguish interdomain motion from intradomain relaxation, we further calculate the RMSD for each domain (Figure 1d,e). It can be seen that both domains undergo initial relaxation within ~125 ns and subsequently stabilize at 2.0 Å (RH domain) and 2.5 Å (kinase domain), presenting small differences between the active and inactive states. The observation indicates that GRK5 maintains stable intradomain folds in the two functional states. Combining the observations from Figure 1b–e, it is reasonable to assume that the overall conformational change is dominated by the relative motion between the RH and kinase domain.

To further characterize the conformational state distribution during the simulations, we concatenate the three 300 ns parallel trajectories and perform a clustering analysis by using the overall RMSD and the Val92–Arg455 interdomain distance as reaction coordinates, as shown in Figure 1f. It can be seen that the inactive and active states present significantly different conformation distributions, further confirming that the interdomain reorientation is the main contributor to the conformational difference between the inactive and active ensembles.

These results also indicate that our simulations can accurately reproduce the critical structural hallmarks reported in distinguishing inactive and active GRK5, such as disruption of the interdomain interactions and the consequent changes in relative domain orientation, thus providing a reliable conformational dataset for subsequent deep learning-based analyses of critical residues.

### 2.2. Unbiased Identification of GRK5 Activation Determinants by Deep Learning

**Figure 2:**
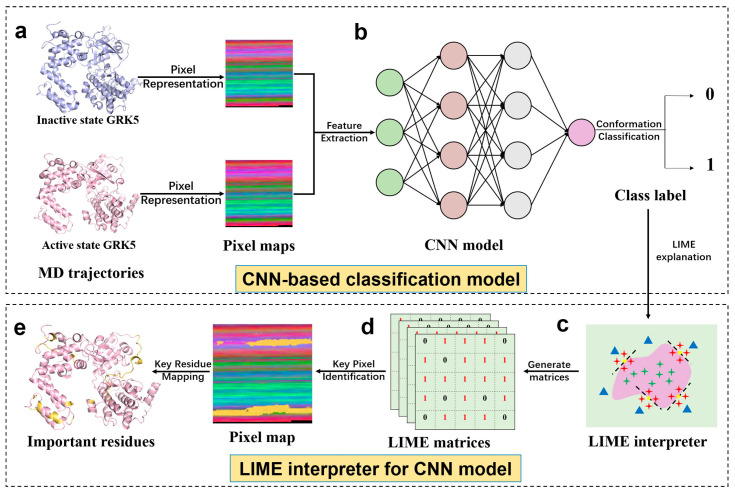
Workflow for GRK5 conformational classification and interpretable residue identification using ICNNMD. (**a**) Atomic coordinates from MD simulation conformations of the inactive and active GRK5 are converted into RGB pixel maps as the CNN input. (**b**) A convolutional neural network (CNN) is trained to classify GRK5 conformations using inactive and active states as class labels. (**c**) Model decisions are interpreted by using the LIME framework. Light green and purple backgrounds denote different classes. For a specific conformation (yellow dot), green crosses and blue triangles indicate positive and negative training samples, while red crosses represent locally perturbed samples generated near the explained conformation. The dashed line indicates the local linear decision boundary. (**d**) LIME matrices. The LIME interpreter generates a matrix with binary values: 1 denotes pixels considered critical to the classification, and 0 denotes those with little influence. (**e**) Projection of important pixels into GRK5 structure to identify important residues. LIME-derived conformation-level importance matrices are ensemble-averaged to identify important pixels (with important residues specially highlighted in yellow in the pixel map), which are then projected onto the protein structure to locate key residues (marked in yellow).

As mentioned above, MD simulations can provide structure and dynamic changes at the atomic level, yet the complex and high dimensional MD trajectory also leads to analytical difficulties. Traditional manual analyses are generally constrained by prior knowledge, for example, mainly concerning important regions and interactions already reported, which easily are overlooked and unreported, being subtle but important structural changes in the complex environment. With the development of artificial intelligence (AI), machine learning as a key facet of AI has been successfully applied in various fields due to its powerful data-mining capabilities, including MD simulations [33,34,35]. However, ML applications in the MD field of proteins mostly involved force field construction and enhanced sampling. Unfortunately, ML focusing on the conformation analyses of proteins has been very limited. To address the absence, our group developed an interpretable deep learning model (ICNNMD) [36] which can accurately classify the conformation states of an MD trajectory by a convolutional neural network (CNN) and a pix image representation, without information loss and complex descriptor calculations. More importantly, it can capture important features deciding conformational classification by constructing the Local Interpretable Model-agnostic Explanations (LIME) interpreter (see Materials and Methods for more descriptions). Thus, in the work, we applied ICNNMD to unbiasedly identify important residues and regions deciding the inactive and active states of GRK5. Specifically, with this framework, the conformations from molecular dynamics (MD) trajectories are first converted into pixel-based feature maps (pixel maps). A classifier based on CNN is then trained to automatically distinguish the active and inactive GRK5 conformations. Then, the LIME interpreter is used to identify key residues deciding the model’s classification task, such that it obtains the important structure features of the GRK5 activation (Figure 2). The five-fold cross-validation achieves 100% classification accuracy, confirming the ability of ICNNMD in classifying the inactive and active conformations.

**Figure 3:**
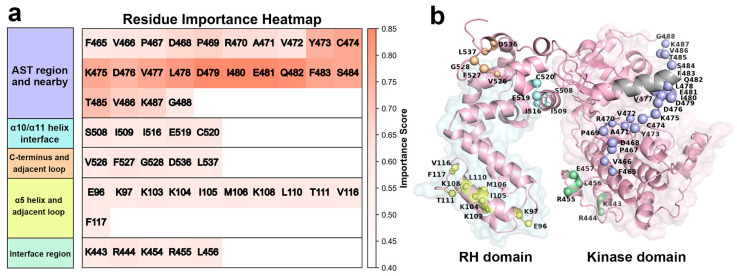
Key residues discriminating the inactive and active states of GRK5, based on the concatenated trajectory of the three parallel trajectories by using interpretable deep learning. (**a**) Heatmap of importance scores for the top 50 residues grouped by structural region. Colors represent normalized importance values. Residue labels are colored consistently with panel A. (**b**) Spatial distribution of the top 50 key residues mapped onto the three-dimensional structure of active GRK5. Residues are shown as Cα spheres, colored according to structural region: blue, active site tether (AST); cyan, α10/α11 helix interface; orange, C-terminus and adjacent loop; yellow, α5 helix and adjacent loops; green, interface region. The disordered αN-helix, absent from the inactive-state structure and excluded from model training, is shown in gray.

The LIME interpreter identifies the top 50 key residues (Figure 3a) with detailed residue indices and regional locations, in which each residue is assigned an importance score ranging from 0 to 1, for which 0 indicates a negligible contribution and 1 represents the highest contribution to the classification. The distribution of these residues is depicted in Figure 3b. It can be seen that these key residues are not randomly distributed, but cluster in specific spatial regions. One part localizes to previously characterized functional regions, including the AST and the RH/kinase domain interface. Specifically, the RH/kinase domain interface involves helix α4 and the C-terminus of helix αJ, which is labeled as the “Interface Region”. The observation clearly shows that our model can capture established activation-related features, demonstrating its rationality and reliability. Interestingly, the other important regions identified involve three less-studied structural modules: disordered C-terminus region, the interface between helices α10 and α11, and helix α5, along with its adjacent loop. Among these, the C-terminus region contains five potential activation-related residues. However, due to its intrinsic structural disorder and high flexibility, this region is not discussed in depth in this study. As a canonical regulatory module for GRK5 activation [30], the interface region contains five key residues identified by ICNNMD. The activation characteristic of the increasing interdomain distance and fluctuations is already described in the previous section (Section 2.1).

Therefore, we focus our discussion on the AST region and the two understudied regions identified by our model as potentially critical for GRK5 activation: the α10/α11 interface and the α5 helix/loop region. Through a detailed analysis of these regions, we aim to elucidate their structural and dynamic characteristics in the activation mechanism and their contribution to global conformational regulation.

#### 2.2.1. Role of the Active Site Tether (AST) in GRK5 Activation

Our interpretable deep learning model reveals a strong enrichment of features within the AST region (residues 468–492) that distinguish the inactive state from the active one (Figure 3). Among the top 50 high-contributing residues identified by the model, a significant proportion (n = 21) are located within this region, exhibiting high importance scores. These results underscore the critical role of the AST region in GRK5 activation dynamics, highlighting it as a high-priority structural hotspot for further mechanistic investigation and inhibitor design.

**Figure 4:**
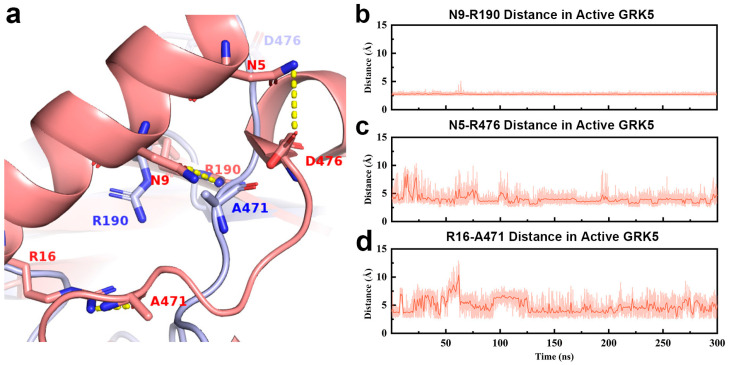
Interactions between αN and kinase domain residues for GRK5. (**a**) Representative interactions between αN and the kinase domain in the active state (pink). Asp476, Arg190, and Ala471 in kinase domain interact with Asn5, Asn9, and Arg16 on the head, middle, and tail of αN, respectively, as indicated by yellow dashed lines. The corresponding positions of these kinase domain residues in the inactive-state structure (blue) are shown for comparison. (**b**–**d**) Time evolution of inter-residues distance in MD simulations for (**b**) Asn9 OD1 and Arg190 NH1, (**c**) Asn5 ND2 and Asp476 OD2, (**d**) Arg16 NH2 and Ala471 O. The results from the other two parallel trajectories are placed in Figure S2 in the Supporting Information.

To elucidate the role of AST in the conformational switch, we compare its conformation and residue interactions between the inactive and active states (Figure 4). Previous studies reported that structural ordering of the AST region is a central hallmark of GRK family activation, with functions extending beyond its canonical role in nucleotide binding [18,19,20,37]. The formation of the αN-helix is recognized as a key step in kinase domain activation [18,38]. In the active state, the formation and stabilization of the αN-helix depend on multiple interactions with the N-lobe and AST region (Figure 4a) [39,40,41]. Accordingly, we analyze inter-residue contact distances between three regions for the active crystal structure of GRK5, showing that the three pair inter-residues present lower values than 4.5 Å (the closest non-hydrogen atoms), such as Asn9 OD1 and Arg190 NH1, Asn5 ND2 and Asp476 OD2, and Arg16 NH2 and Ala471 O, in which Ala471 and Asp476 locate in the AST region, while Asn9, Arg16, and Asn5 distribute over the αN-helix. Arg190 belongs to the N-lobe. Comprehensive prior studies [42,43,44] indicated that the 4.5 Å contact distance generally encompass various interactions between residues like polar, nonpolar, and hydrogen bonds. Thus, we monitor the change in the three distances during 300 ns simulation, as shown in Figure 4. Our MD trajectory analysis reveals that the Asn9–Arg190 interaction remains highly stable, as evidenced by the stable distance that is significantly lower than 4.5 A (Figure 4b). The distances of the Asn5–Asp476 and Arg16–Ala471 pairs exhibit initial fluctuations but are subsequently almost stabilized within 4.5 Å (Figure 4c,d). These simulation results support stabilizing interactions between AST and αN in the active state which promote the formation of the αN-helix. In contrast, the AST region in the inactive state displays a markedly different conformation. Although local ordering is observed in some inactive conformations, the spatial positions of Ala471 and Asp476 are substantially shifted relative to the active state, preventing effective interactions with αN and thus failing to stabilize the helical structure (Figure 4a). This indicates a fundamental reorganization of the AST–αN interaction landscape between states: specific active state contacts stabilize αN, while in the inactive state, the AST region lacks these contacts. 

**Figure 5:**
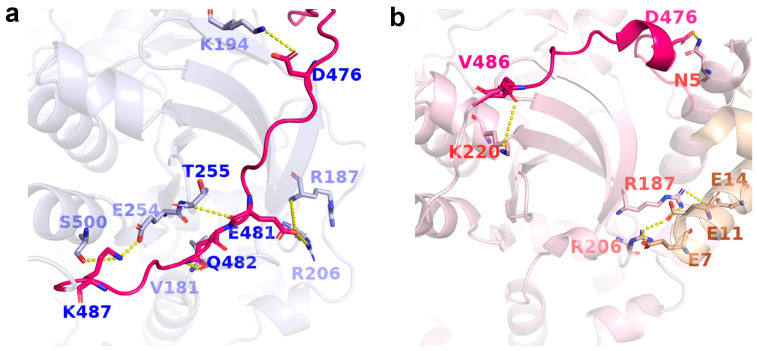
Key interactions between the AST and the kinase domain N-lobe of GRK5. (**a**) Inactive-state GRK5. (**b**) Active-state GRK5. The AST region is highlighted in magenta. Key residues are shown as sticks, with representative interactions indicated by yellow dashed lines. In the active state structure, CaM-associated residues are highlighted in wheat.

In the inactive state, the AST region also engages a unique set of contacts that anchor the kinase domain in an open conformation. Consistent with previous structural studies [18,45,46], specific rearrangements of the AST loop can directly promote closure of the kinase lobe, leading to full activation. Specifically, in our simulations of the inactive state, the N-lobe-tethering segment of AST (residues 474–492; e.g., Asp476, Glu481, Gln482) forms interactions with residues in the kinase domain N-lobe (e.g., Ser500 on αK, and Arg187, Lys194, Thr255 on the antiparallel β-sheet), anchoring it within a hydrophobic pocket formed by the β1–β5 sheet (Figure 5a). The average distances for most of these residue pairs increase during the simulations relative to the crystal structure (Table S1, Figure S3). Except for the relatively stable Glu481–Thr255 and Gln482–Val181 pairs, most residue pairs show substantial fluctuations without reaching a steady state within 300 ns, indicating a feature of “local anchoring but overall dynamic instability” for the inactive state AST. In contrast, interactions involving the AST in the active state are more localized. For instance, only Val486 forms a relatively stable interaction with Lys220 on the αB helix (Figure S4), securing the AST along the lateral surface of the kinase domain N-lobe (Figure 5b). Collectively, these observations suggest that AST-mediated anchoring in the inactive state helps restrict relative motion between the kinase domain N-lobe and C-lobe, disfavoring transition to the closed, active conformation.

Functionally relevant rearrangements at this interface are also likely to impact upstream binding events. The AST region directly participates in GRK–GPCR interactions [19,20], while the kinase domain N-lobe (particularly Arg187 and Arg206 on β1–β3) forms a critical CaM-binding interface [18], as evidenced by Figure 5b. In inactive state trajectories, Glu481 of the AST forms transient interactions with Arg187 and Arg206 (Figure S3b,c, Table S1). These contacts not only help anchor the AST to the β1–β5 antiparallel β-sheet but also spatially occupy the CaM-binding site (Figure 5a) [47,48,49].

Collectively, integrating unbiased residue importance mapping from deep learning with conformational analysis from MD simulations demonstrates that the AST region undergoes clear state-dependent conformational rearrangement and interaction changes during GRK5 activation, highlighting its critical regulatory role. This provides strong candidate sites for subsequent experimental validation and pharmacological intervention. Notably, prior studies have explored the AST region as a target for high selectivity inhibitor design, for example, by targeting the non-conserved Cys474 with covalent inhibitors to stabilize specific conformations (e.g., the inactive state) and achieve selective GRK5 modulation [12]. Furthermore, a recent work further demonstrated that novel non-covalent inhibitors can also achieve low nanomolar potency and exceptional selectivity by interacting with the AST loop [50,51]. Thus, targeting the AST region is a feasible and promising strategy for developing inhibitors with enhanced selectivity and state specificity.

#### 2.2.2. Mechanisms of α10/α11 Interface and NLBD Coupling in GRK5 Activation

The explainable deep learning model further reveals that Glu519 and Cys520, located at the junction of the α10/α11 helices, significantly contribute to distinguishing between the inactive and active states of GRK5 (Figure 3). Their importance scores are second only to those of the AST region. This entirely data-driven, hypothesis-free result suggests that this region, despite receiving limited attention in previous studies, may play a pivotal role in GRK5 activation. Notably, prior studies considered the α10 helix as a dynamic hinge mediating the relative rotation between the RH and kinase domain [30]. The identification of key residues in the immediate vicinity of this functional hinge implies that this region is likely involved in regulating the global conformational state of GRK5.

As Glu519 and Cys520 are situated at the interface of the α10 and α11 helices, their functional effects are likely mediated by conformational rearrangements within this region. Molecular dynamics simulations indicate that state-dependent conformational adjustments in this region influence the spatial orientation and interaction of Glu523 located at the N-terminus of helix α11. Consequently, due to its spatial proximity to the identified Glu519/Cys520 pair and its position downstream of the α10/α11 hinge, Glu523 is selected as a representative residue for subsequent structural and dynamic analyses.

**Figure 6:**
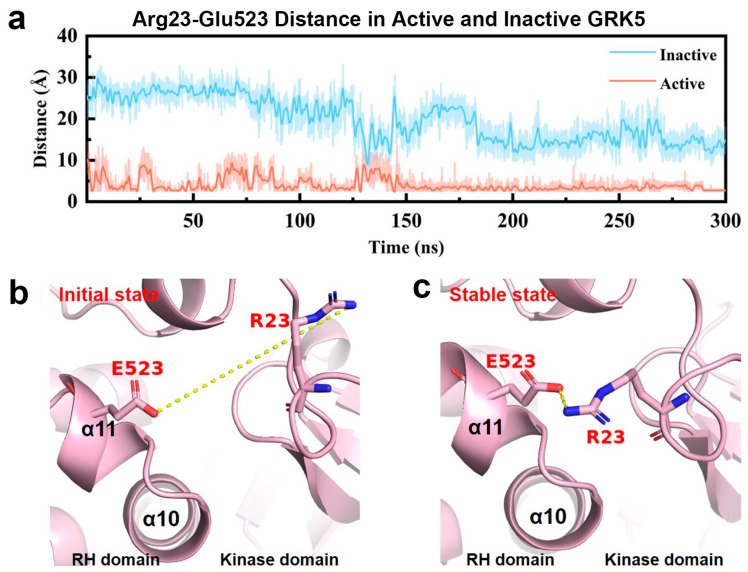
Dynamics change in the Glu523-Arg23 distance in the active state of GRK5. (**a**) Time evolution of the closest non-hydrogen atomic distance between the Glu523 OE2 and the Arg23 NH2 atoms in the inactive (blue) and active (red) states. The results from the other two parallel trajectories are placed in Figure S5. (**b**) Initial conformations of Arg23 and Glu523 prior to active state simulations. (**c**) Stabilized conformation of Arg23 and Glu523 observed during active state simulations.

As reflected by Figure 6, the Arg23–Glu523 distance (calculated by the closest non-hydrogen atoms) in the active state remains stable with an average of 3.0 ± 0.6 Å, after ~150 ns of conformational relaxation, indicating its dynamic stability. In contrast, the Arg23–Glu523 distance in the inactive state simulations exhibits large and stochastic fluctuations due to being disordered, and, in turn, not forming stable contacts (Figure 6a). These findings demonstrate that the Glu523–Arg23 interaction is a specific structural feature of the active state.

Further structural analysis reveals that the formation of this active-state-specific interaction directly depends on the ordering of the αN-helix. In the active state, the ordering of the αN-helix promotes the ordering of the adjacent NLBD, thereby providing the necessary structural prerequisite for the interaction between Arg23 (NLBD) and Glu523 (α11) (Figure 6b,c). Conversely, in the inactive state, the high disorder of both the αN-helix and the NLBD results in a far distance from the α10/α11 interface, thereby preventing the establishment of stable contact (Figure 6a). This result links the stability of the AST–αN interface (observed in Section 2.2.1) with the newly identified NLBD–α11 interface, revealing a long-range conformational coupling that spans multiple domains during GRK5 activation.

Based on these observations, we propose that in the active state, the ordering of the αN-helix not only stabilizes the kinase domain itself but also restricts the conformational freedom of the NLBD, enabling specific contact with helix α11. As this interaction occurs near the α10/α11 dynamic hinge, it likely modulates the hinge’s conformation state, indirectly constraining the range of motion of the RH domain and helping to maintain the interdomain geometry required for activation. From a structure–function perspective, the observation that the NLBD–α10/α11 interface is stable only in the active state and is mediated by some specific residues suggests that this feature is unique to the active state. This finding further indicates that the regulatory role of the αN-helix in GRK5 activation is not limited to the kinase domain interior but extends to the more distal α10/α11 interface.

#### 2.2.3. Conformational Role of the α5 Helix and Adjacent Loop in GRK5 Activation

Beyond the AST and α10/α11 regions, our interpretable deep learning model also identifies the α5 helix and its adjacent loop as a structural hotspot, comprising 11 key residues (Figure 3). Notably, the average importance score of this region surpasses that of the canonical interface region. This unbiased computational finding underscores the region’s substantial conformational contribution to the GRK5 activation.

**Figure 7:**
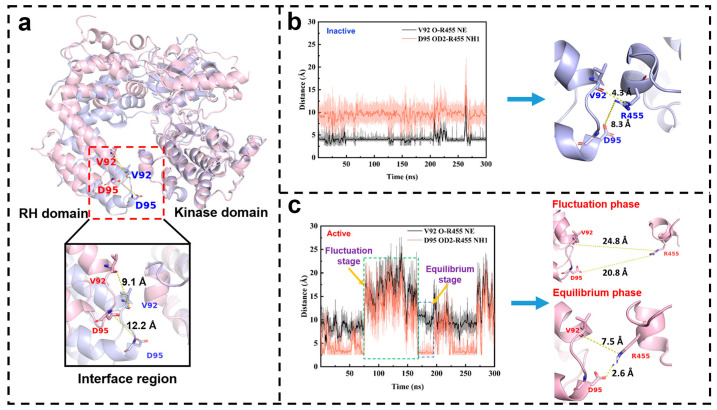
Activation-induced changes at the α5 helix and interface region residues in GRK5, derived from one representative trajectory. (**a**) Structural superposition of inactive (light blue) and active (light pink) GRK5, with a detailed lateral view of the interface region. Val92 and Asp95 are shown as sticks. (**b**) The closest non-hydrogen atomic distances between the Val92 O and Arg455 NE atoms (black) and between the Asp95 OD2 and Arg455 NH1 atoms (red) in inactive GRK5. Representative conformation snapshots are shown on the right. (**c**) The closest non-hydrogen atomic distances between the Val92 O and Arg455 NE atoms (black), and the Asp95 OD2 and Arg455 NH1 atoms (red) in the active GRK5. Fluctuation and equilibrium phases are highlighted by green and blue dashed boxes, respectively. Representative snapshots of these phases are shown on the right. The results from the other two parallel trajectories are placed in Figure S6, in Supporting Information.

To elucidate the underlying mechanism, we compare the RH domain conformations between the active and inactive states. Structural superposition of the inactive and active states reveals that the activation involves not only the disruption of inter-domain interactions but also a major out-of-plane “untwisting” of the RH domain relative to the kinase domain (Figure 7a). This motion is most pronounced at the α5 helix, where the representative residue Asp95 undergoes a displacement of 12.2 Å, exceeding the 9.1 Å shift in the interface residue Val92. This observation indicates that the α5 region experiences more pronounced conformational adjustments during the overall rearrangement, suggesting that it should play an important role in the untwisting motion. This structural insight supports the interpretable result from our deep learning model.

Furthermore, we observe a dynamic, state-specific interaction between Asp95 (on helix α5) and Arg455 (in the kinase domain), the variation of which follows the same trend as the canonical Val92–Arg455 interface distance (Figure 7b,c). In the inactive state, the Asp95–Arg455 distance is consistently larger than the Val92–Arg455 distance (Figure 7b), consistent with the twisted RH geometry. Upon activation, this trend reverses: the Asp95–Arg455 distance is predominantly smaller than that of Val92–Arg455, and periodically approaches ~3 Å, implying an interaction that is absent in the inactive state (Figure 7c).

This dynamic behavior gives rise to two conformational phases in the active state: a fluctuation phase and an equilibrium phase (Figure 7c). During the equilibrium phase (when the Asp95–Arg455 interaction forms), the interaction effectively pulls the α5 helix toward the kinase domain, stabilizing the untwisted RH conformation. Conversely, in the fluctuation phase (when this contact breaks), the domain remains untwisted but is less spatially restrained. Thus, the α5 region refines and dynamically stabilizes the active state via this specific, reversible interaction.

Combining observations from the machine learning model, it can be assumed that the α5 helix and its adjacent loop region not only play a conformational regulatory role in GRK5 activation but also facilitate the formation of the untwisted RH domain conformation by establishing new, activation-specific interactions with the kinase domain. This mechanism diverges from the traditional disruption of the interdomain interactions and offers fresh perspectives on the interdomain movements underlying GRK activation.

### 2.3. Dynamic Cross-Correlation Analysis

To gain insights into changes in residue–residue interactions upon activation at the dynamic level, we calculate the dynamic cross-correlation matrix (DCCM) from the concatenated MD trajectory of the three parallel simulations for inactive and active GRK5. DCCM can quantify linear correlations between Cα atom fluctuations (coefficients ranging from −1 to +1, corresponding to completely anticorrelated and completely correlated motions, respectively), thereby revealing dynamic changes in the coupling among structural regions [52].

**Figure 8:**
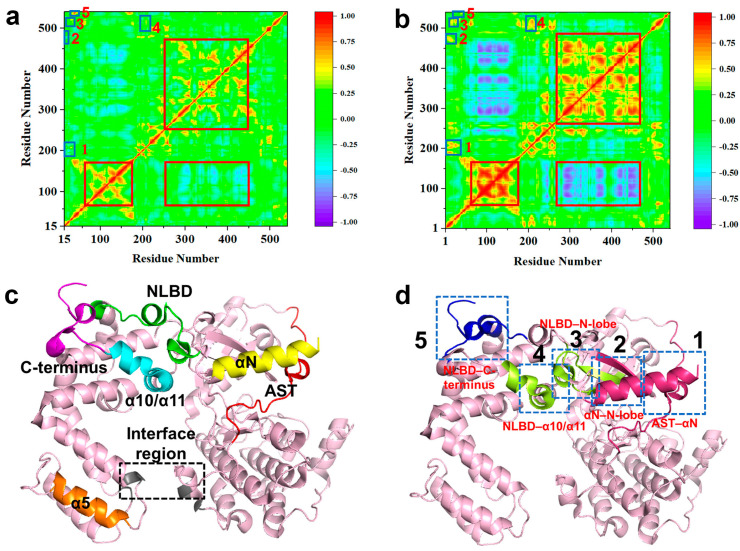
DCCM analysis of GRK5 and locations of key regions from the concatenated trajectory of three parallel 300 ns simulations. DCCM plots of (**a**) inactive and (**b**) active GRK5, with the red box highlighting correlations within the RH bundle subdomain and with the kinase domain C-lobe in the inactive state. Blue boxes mark other regions showing pronounced differences. (**c**) Key GRK5 regions: αN (residues 1–17, yellow), NLBD (22–39, green), α5 (97–122, orange), AST (469–478, red), α10/α11 (513–524, sky blue), and C-terminus/interface (533–542, purple). (**d**) Blue box 1 represents αN and AST region, blue box 2 represents αN and kinase domain N-lobe, blue box 3 represents NLBD and kinase domain N-lobe, blue box 4 represents NLBD and α10/α11, blue box 5 represents NLBD and C-terminus, respectively. Dynamically correlated sets identified from DCCM analysis: αN-NLBD-N-lobe-AST (pink, blue boxes 1–2), NLBD–α10/α11 (yellow-green, blue boxes 3–4), NLBD-C-terminus (blue, blue box 5).

A comparison between Figure 8a,b clearly shows that the correlated motion between the RH bundle subdomain and the kinase domain C-lobe is markedly enhanced in the active state relative to the inactive state, in line with the frequent interdomain separation fluctuations observed in the simulations. In addition, we identify five local regions where correlations change substantially upon activation, as illustrated by the blue boxes labeled as 1–5 in Figure 8a,b.

#### 2.3.1. Formation of Cooperative Motions Between Four Regions (αN, NLBD, Kinase Domain N-Lobe and AST)

DCCM analysis reveals a newly emerged set of positive correlations in the active state involving the αN-helix (residues 1–16), the N-terminal segment of the NLBD (residues 22–27), the kinase N-lobe β-sheets (residues 187–192, 200–215), and the AST region (residues 467–488) (Figure 8a,b, blue boxes 1, 2 and 3). While these regions are predominantly weakly or negatively correlated in the inactive state, they transition to significant positive correlations upon activation, with coefficients reaching up to 0.77. This provides dynamic evidence supporting the structural analysis that the ordering of the αN-helix and the AST region stabilizes the spatial positioning of the NLBD (vide Section 2.2.2). Consequently, these regions no longer move as relatively independent units, but participate in a cooperative conformation mode, establishing dynamic correlation facilitating cross-domain signal transduction.

#### 2.3.2. Formation of Cooperative Motions Between NLBD, α10/α11, and Kinase Domain

Furthermore, DCCM analysis provides independent dynamic support for the aforementioned newly identified “NLBD–α10/α11” interaction interface (blue boxes 3 and 4 in Figure 8a,b). In the active state, the NLBD and the α10/α11 helices (residues 513–524) exhibit significant positive correlated motion (correlation ~0.65), which is absent in the inactive state. Simultaneously, the correlated motion between the NLBD and the kinase domain N-lobe is also markedly enhanced.

These results indicate that NLBD in the active state is no longer a dynamically independent segment but forms cooperative motions between the α10/α11 hinge and the kinase domain. Combining with the structural evidence of α10/α11 as a key conformational hub (Section 2.2.2), this cross-domain synergistic pattern likely plays a central role in stabilizing and maintaining the active state conformation.

#### 2.3.3. Decoupling Motion Between NLBD and C-Terminus

In the active state, as NLBD incorporates into the cooperative motion pattern centered on the α10/α11 helices and the kinase domain, its dynamic coupling with the C-terminus region, which is observed in the inactive state, undergoes a significant change. DCCM analysis reveals that in the inactive state, the C-terminal segment of the NLBD (residues 35–42) and the C-terminus (residues 532–542) exhibit highly correlated motion (correlation coefficient ~0.8), indicating that these two highly flexible regions share a synchronized fluctuation pattern in the inactive conformation (Figure 8a,b, blue box 5).

In contrast, this correlation significantly decreases to 0.3 in the active state, indicating a decoupling of the concerted motion between the NLBD and the C-terminus during activation. Given the high conformational disorder of the C-terminus in GRK5 [53,54,55,56,57], this change does not simply reflect an overall increase or decrease in flexibility, but more likely represents a reorganization of the dynamic couplings of the NLBD during activation. Combining this finding with the cross-domain coordinated pattern involving the NLBD, α10/α11, and the kinase domain described in Section 2.3.2, we infer that the decoupling of the NLBD–C-terminus-correlated motion is related to the NLBD–α10/α11 interactions.

### 2.4. Global Motion Analysis of GRK5 Activation

To further validate the dynamic characteristics of critical conformational rearrangements during GRK5 activation at the global motion level, we perform principal component analysis (PCA) on the concatenated trajectory of three parallel 300 ns simulations, both for the inactive and active GRK5. This method extracts dominant collective motion modes from complex atomic fluctuations, thereby revealing the primary directional features of conformational changes [56,57,58]. This analysis complements the pairwise correlations identified by DCCM.

**Figure 9:**
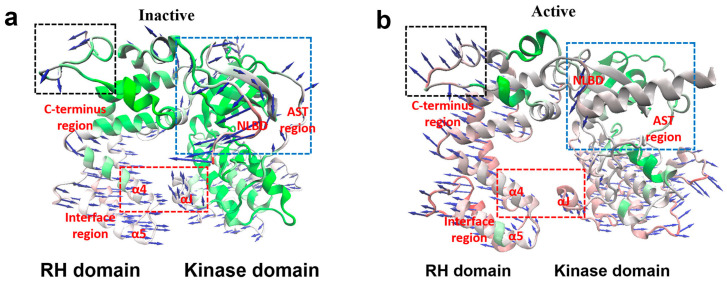
Principal component analysis (PCA) of collective motions in GRK5 based on the concatenated trajectory of three 300 ns parallel simulations. (**a**) Projection of the MD trajectory for the inactive GRK5 onto the first principal component (PC1). The color map encodes the amplitude of residue motions along PC1 (green: low; white: medium; red: high). Functionally relevant regions are highlighted with dashed boxes. Purple arrows indicate the mean displacement vectors of Cα atoms along PC1, with arrow lengths proportional to the magnitude of motion. (**b**) Projection of the MD trajectory for the active GRK5 onto PC1, presented with the same color mapping and vector representation as in panel (**a**).

PCA clearly reveals large-scale domain separation upon activation. As shown in Figure 9a,b, the RH bundle subdomain and the kinase domain C-lobe exhibit contrasting motion patterns during MD simulation. Specifically, in the active state, the two structural units display larger amplitudes and move in opposing directions (Figure 9b), whereas motions in the inactive state are notably restricted in both amplitude and directionality (Figure 9a). This global motion aligns well with increased interdomain distance (Section 2.1) and enhanced cooperative rearrangement (Section 2.3), confirming that GRK5 activation involves pronounced displacement between the RH and kinase domain.

For the most important region revealed by the machine learning model, in the inactive state, the AST region and kinase domain N-lobe display divergent motion trends along the dominant mode (Figure 9a). This observation is consistent with the “unstable anchoring” state described in Section 2.2.1 and further supports the role of this region in imposing conformational constraints on the inactive ensemble.

Moreover, PCA indicates that in the active state, NLBD exhibits significantly reduced motion amplitude along the principal mode relative to the inactive state (Figure 9b). This reflects a transition from a highly flexible segment to a more conformationally restrained state upon αN-helix ordering, aligning well with the conclusion that the NLBD becomes integrated into the activation-related dynamic regulatory framework (Section 2.2.1 and Section 2.3).

In addition, PCA provides further dynamic insights into the principal motion directions of the interface region. In the inactive state, the α4/α5 helices and the αJ helix exhibit an approximately orthogonal relationship in their dominant motions, which is conducive to maintaining conformational stability at the interface region (Figure 9a). In contrast, in the active state, the dominant motion directions of the α4 and αJ helices rearrange into distinctly opposing motions. This pattern is consistent with the activation features of disrupted interdomain interactions and domain separation (Figure 9b). Notably, the α5 helix in the active state shows a dominant motion tendency directed toward the αJ helix. This collective motion direction aligns well with the mechanism proposed in Section 2.2.3, where the α5 region participates in the “untwisting” and repositioning of the RH domain by forming new, transient interactions.

For the C-terminus region of GRK5, which is also identified by our ML model as a key region involved in activation, the PCA results show an increased amplitude of motion in the active state. This trend corresponds to the correlated motion associated with the C-terminus observed in the DCCM analysis (Section 2.3.3). Given the proximity of this region to the CaM binding site, this change in dynamic characteristics may be related to CaM recognition in the active state.

Collectively, from the perspective of global motion patterns, PCA provides independent yet consistent dynamic support for the interdomain separation, the “untwisting” conformational transition, and the dynamic rearrangement of key functional modules during GRK5 activation.

## 3. Materials and Methods

### 3.1. System Preparation

The crystal structures of the inactive GRK5–sangivamycin complex (PDB ID: 4TNB) and the active GRK5–CaM complex (PDB ID: 6PJX) were obtained from the Protein Data Bank. All crystallographic waters and ions were removed using PyMOL (version 3.1.6.1, retaining only the protein–ligand complexes. System setup, including ligand retention and parametrization, solvation, and ion addition, was conducted using the CHARMM-GUI web server [59]. The protonation states of titratable residues were assigned based on their dominant forms at pH 7.0. Each system was solvated with the TIP3P water model in a rectangular box, ensuring a minimum clearance of 12 Å between the protein surface and the box boundaries. The systems were neutralized and adjusted to 0.15 M NaCl concentration by adding appropriate amounts of Na^+^ and Cl^−^ ions. The protein and ions were parameterized using the CHARMM36 force field [60,61,62], while the ligands were modeled using the CHARMM General Force Field (CGenFF) [63].

### 3.2. Molecular Dynamics Simulations

Molecular dynamics (MD) simulations were performed using AMBER18 software. The simulation protocol for each system proceeded in three stages: energy minimization, heating, and equilibration.

First, a three-step energy minimization procedure was employed to relax the system gradually. In the first step, minimization was restricted to solvent molecules and ions, while the protein and ligand were subjected to a harmonic positional restraint with a force constant of 100 kcal mol^−1^ Å^−2^. In the second step, the restraints on all atoms of the protein and ligand were reduced to 20 kcal mol^−1^ Å^−2^ to allow for overall structural relaxation. In the third step, restraints were applied only to the protein backbone and ligand heavy atoms (10 kcal mol^−1^ Å^−2^), allowing the side chains and solvent environment to be fully optimized. Each minimization step consisted of 20,000 steps.

After minimization, the system was heated from 0 K to 310 K over 250 ps using a Langevin thermostat. This process was conducted in two phases (0 K to 100 K over 125 ps, followed by 100 K to 310 K over 125 ps), with positional restraints of 10 kcal mol^−1^ Å^−2^ maintained on the protein and ligand throughout. Subsequently, a seven-step equilibration was performed at 310 K (1 ns per step), during which the positional restraints were gradually reduced to zero to ensure full system relaxation. The cut off of the non-bonded interactions was set to 10 Å and the particle mesh Ewald (PME) method was applied to treat the electrostatic interactions.

For production runs, three parallel 300 ns unrestrained MD simulations were conducted for each state, starting from the final equilibrated structure. To ensure statistical independence, initial atomic velocities were assigned using different random seeds for each replicate. Trajectory analysis, including principal component analysis (PCA), was performed with CPPTRAJ in AmberTools, and structural visualizations were generated with VMD [64,65].

### 3.3. The Interpretable Convolutional Neural Network for Molecular Dynamics (ICNNMD)

In this study, we employed the ICNNMD framework, a deep learning approach previously developed by our group [35]. This framework utilizes a pixel map representation of protein conformations and leverages the powerful feature extraction capabilities of convolutional neural networks (CNNs) to accurately classify conformations associated with distinct functional states from MD trajectories. Furthermore, ICNNMD also incorporates the locally linear approximation paradigm [66] to implement a LIME interpreter. This approach addresses the ‘black box’ nature of deep learning models, enabling the identification of key residues that influence the classification results.

For the MD simulations, we performed production runs on both the inactive and active GRK5 systems, each generating three parallel 300 ns datasets. We extracted one conformation every 30 ps, yielding a dataset of 10,000 conformations per trajectory. By concatenating the three parallel trajectories, we obtained 30,000 conformations for each state (in total, 60,000 conformations of two states for model training). To ensure input consistency and eliminate translational and rotational differences, all conformations were aligned via rigid-body fitting, followed by solvent molecule and ion removal to retain only protein atoms. The 3D coordinates of each atom were then transformed via matrix transformation into pixels in the RGB color space, generating standardized pixel maps as model inputs. This representation strategy maximizes the preservation of spatial structural information while avoiding the potential information loss associated with manual feature extraction.

The classification module of ICNNMD is constructed based on a convolutional neural network (CNN). The CNN model comprises four convolutional layers: the first two layers use 32 kernels each, and the subsequent two use 64 kernels each. All layers employ the ReLU activation function to extract features from pixel map representations. A max-pooling layer with 2 × 2 filters and a dropout rate of 0.25 follows every two convolutional layers to reduce dimensionality and prevent overfitting. The feature maps extracted by convolution and pooling are then fed into a fully connected neural network for classification. The first fully connected layer contains 512 neurons activated by ReLU, while the output layer contains 2 neurons using the softmax function for binary classification. The dropout rate for the fully connected layers was set to 0.5 to improve the model’s generalization.

Model training was conducted using five-fold cross-validation. Given that the trajectory data are temporally correlated, each trajectory was divided into 10 groups in chronological order, and each segment was further evenly split into five folds. In each training iteration, one fold from each group was selected as the validation set, while the remaining four folds were combined as the training set. This process was repeated five times to construct a 5-fold cross-validation set. For model optimization, categorical cross-entropy was employed as the loss function. Classification performance was evaluated using accuracy, calculated as follows:

(1) $$Accuracy=\frac{\mathrm{TP}\;+\;\mathrm{TN}}{\mathrm{TP}\;+\;\mathrm{FN}\;+\;\mathrm{FP}\;+\;\mathrm{TN}}$$

where TP represents the number of true positive samples predicted as positive by the model, FP represents the number of true negative samples predicted as positive, FN represents the number of true positive samples predicted as negative, and TN represents the number of true negative samples predicted as negative.

To further explain the classification basis and identify key residues, the LIME interpreter was utilized to identify key residues governing the classification results. LIME is a local surrogate model that explains classifier predictions via local linear approximation. Specifically, for each conformation to be explained, LIME generates perturbed samples within the local neighborhood of the target conformation and weights them based on their similarity to the original instance. In this study, we generated 1000 perturbed samples to explain a single conformation, using Euclidean distances to quantify the proximity between the perturbed samples and the original conformation. Subsequently, a linear model was trained to fit the CNN’s classification output on the perturbed dataset. For a single conformation, the linear model assigns a binary importance score to each pixel (atom), indicating its contribution to the classification decision (0 for insignificant, 1 for significant). The overall importance score for each residue was calculated by averaging the scores of its constituent atoms across all analyzed conformations. The resulting averaged scores range from 0 to 1, with a higher score indicating a greater contribution to the classification decision. Considering the large number of residues in the GRK5 system, the top 50 residues with the highest importance scores were identified as key residues for subsequent structural and functional analyses.

### 3.4. Dynamic Cross-Correlation Matrix (DCCM) Analysis

The dynamic cross-correlation matrix (DCCM) is an essential tool for gaining deeper insights into protein dynamics [67]. It reveals how different regions of the protein move relative to each other over time by calculating the displacement correlations of atoms or residues along the MD trajectory. The cross-correlation coefficient of the position vectors is calculated using the following formula:

(2) $$C_{ij}=\frac{\bar{\left(r_{i}-{\bar{r}}_{i}\right)\left(r_{j}-{\bar{r}}_{j}\right)}}{\sqrt{\bar{\left(r_{i}^{2}-{\bar{r}}_{i}^{2}\right)\left(r_{j}^{2}-{\bar{r}}_{j}^{2}\right)}}}$$

where *i* and *j* represent the indices of the atoms (or the centers of mass of the residues), and *r_i_* and *r_j_* are their corresponding position vectors. The correlation coefficient *C_ij_* ranges from −1.0 to 1.0, where 1.0 indicates perfectly correlated motion and −1.0 indicates perfectly anti-correlated motion. All DCCM calculations were performed using Wordom software (version 0.22-rc3) [68].

## 4. Conclusions

In this study, we have combined molecular dynamics simulations with an interpretable deep learning framework to unbiasedly capture the activation features of GRK5 involving structures and dynamics, which can help to elucidate its activation mechanism.

Our results demonstrate that GRK5 activation involves the disruption of canonical interdomain interface interactions, commonly referred to as the “ionic lock,” together with a pronounced reorganization of the kinase and RH domain. Importantly, the interpretable deep learning model has automatically identified the AST region as the most critical feature differentiating inactive and active states, without relying on prior assumptions. Dynamic analysis has further revealed that AST stabilizes the αN-helix and promotes kinase domain closure in the active state, while its distinct “anchoring” conformation in the inactive state restricts these activation-associated motions. These mechanistic insights establish the AST as a central conformational switch and provide a conceptual framework that could guide the rational design of state-specific modulators targeting GRK5.

Beyond the important AST, our model uncovers two previously underappreciated regulatory regions that contribute to GRK5 activation. One is the interface between helices α10 and α11, where a stable NLBD–α11 interaction emerges in the active state and modulates the central hinge to support the active conformation. The second involves the α5 helix and its adjacent loop, which drive the RH domain transition from a twisted to an untwisted conformation through dynamic interactions with the kinase domain. These results also confirm that combining MD simulations with machine learning can reveal subtle, non-obvious regulatory elements outside the canonical active site.

Conformational dynamics analyses from dynamic cross-correlation matrices (DCCM) and principal component analysis (PCA) reveal that the GRK5 activation is regulated by allosteric couplings between the AST, αN-helix, kinase domain N-lobe, NLBD, and the α10/α11 hinge, underscoring the collective, global nature of activation rather than a localized structural rearrangement.

Collectively, the work identifies important residues, dynamic switches and regulatory hubs that govern GRK5 activation, thus advancing the fundamental understanding of GRK5 activation. Also, the work clearly shows the capacity of machine learning in capturing critical structure features involving function, thus providing a methodological guideline for studying protein structure and function.

## Data Availability

The original contributions presented in this study are included in the article/Supplementary Material. Further inquiries can be directed to the corresponding authors.

## Supplementary Materials

The following supporting information can be downloaded at: https://www.mdpi.com/article/10.3390/ijms27073329/s1.

## Abbreviations

GRK5	G Protein-Coupled Receptor Kinase 5
AST	Active Site Tether
NLBD	N-Terminal Lipid-Binding Domain
GPCR	G Protein-Coupled Receptor
MD simulation	Molecular Dynamics Simulation
ICNNMD	Interpretable Convolutional Neural Network for Molecular Dynamics
PCA	Principal Component Analysis
DCCM	Dynamic Cross-Correlation Matrix
RMSD	Root-Mean-Square Deviation
LIME	Local Interpretable Model-Agnostic Explanations
