# Supplementary material for: Revealing GRK5 Activation Features by Interpretable Machine Learning and Molecular Dynamics Simulation
Source: Int J Mol Sci. 2026 Apr 7;27(7):3329. doi: 10.3390/ijms27073329 (PMC13072926; doi:10.3390/ijms27073329)
Supplement: Supplementary file 1 [file ijms-27-03329-s001.zip › ijms-4167581-supplementary.pdf]

Revealing GRK5 Activation Features by Interpretable Machine Learning and Molecular Dynamics Simulation

Yuanpeng Song[1], Ming Kong[1], Fuhui Zhang[2,*],Xuemei Pu[1,*]

[1] College of Chemistry, Sichuan University, Chengdu 610064, China

[2] Graduate School of Sichuan University, Chengdu, Sichuan, 610200, China

[*] Correspondence: zhangfuhui@scu.edu.cn (F.Z.); xmpuscu@scu.edu.cn (X.P.)

Supplementary Materials

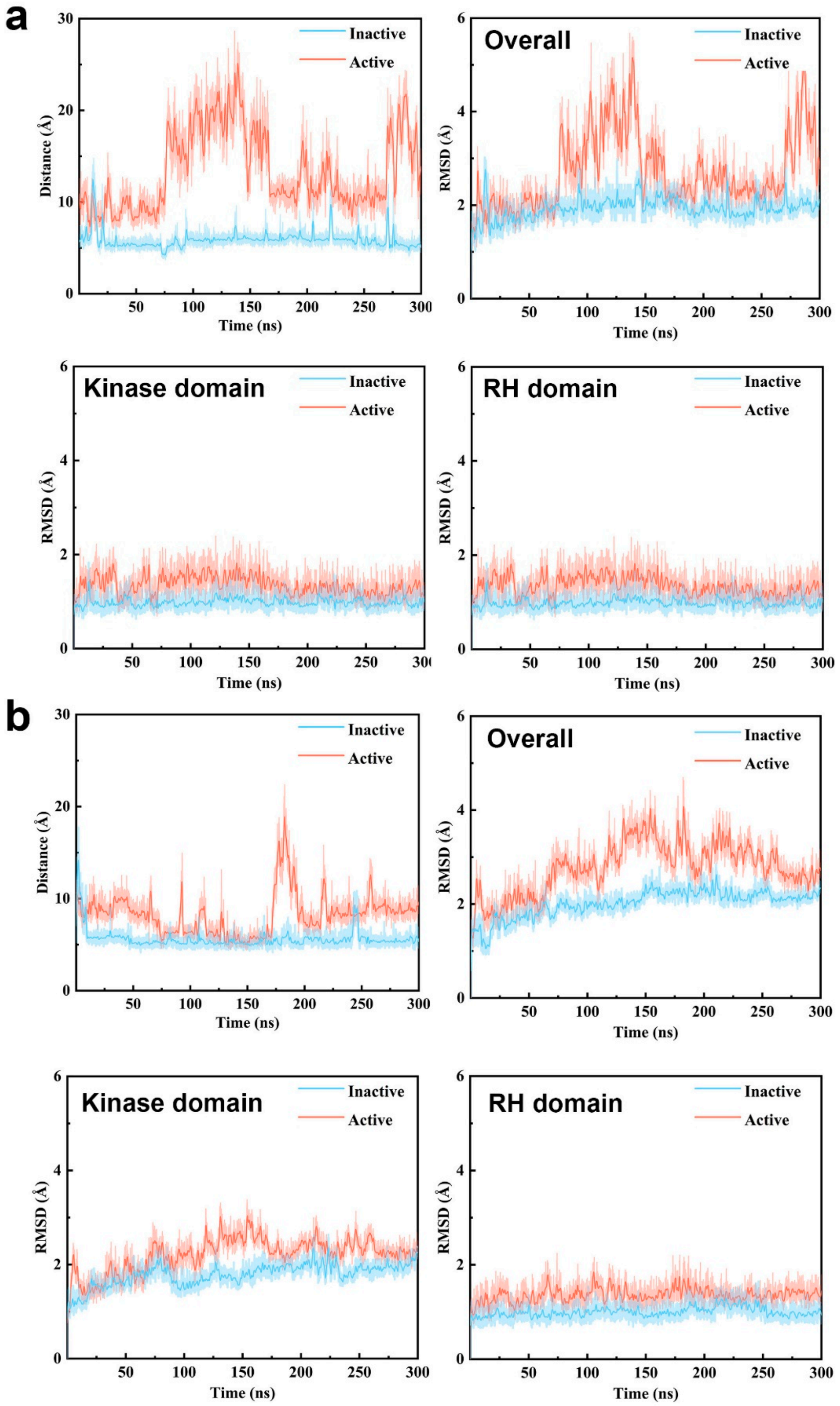

Figure S1. Structural characteristics of inactive and active GRK5 states for the second (a) and the third (b) parallel 300-ns trajectories. The inactive and active states are colored blue and red, respectively. Overall indicates the overall structure of GRK5. All RMSDs were calculated with respect to the initial structure.

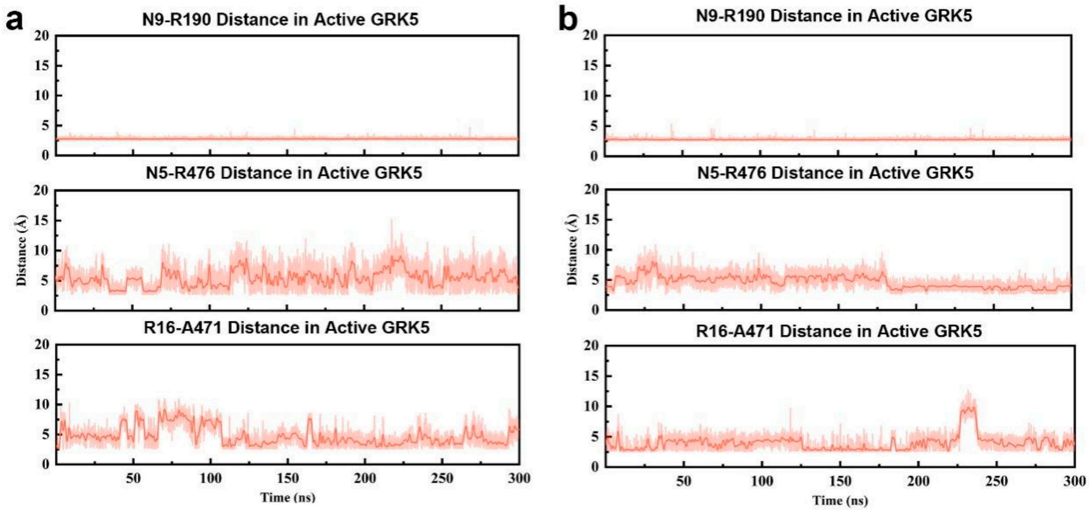

Figure S2. Time evolution of inter-residue distances between αN and kinase domain residues for GRK5 for the second parallel 300-ns trajectory (a) and the third parallel 300-ns trajectory (b). Three inter-residue distances involve Asn9 OD1 and Arg190 NH1, Asn5 ND2 and Asp476 OD2, and Arg16 NH2 and Ala471 O in the active state.

Table S1. Residues distances between the AST region and the kinase domain N-lobe in inactive GRK5 state, including the initial distances observed in the crystal structure and the average distances over three 300-ns parallel MD trajectories.

Interaction	Distance in crystal structure (Å)	Average Distance in MD (Å)
Asp476-Lys194	4.1	9.2
Glu481-Arg187	5.5	8.2
Glu481-Arg206	8.5	9.3
Glu481-Thr255	4.8	5.2
Gln482-Val181	3.8	4.4
Thr485-Glu254	3.4	5.1
Lys487–Lys254	2.7	6.1
Lys487-Ser500	4.3	7.4

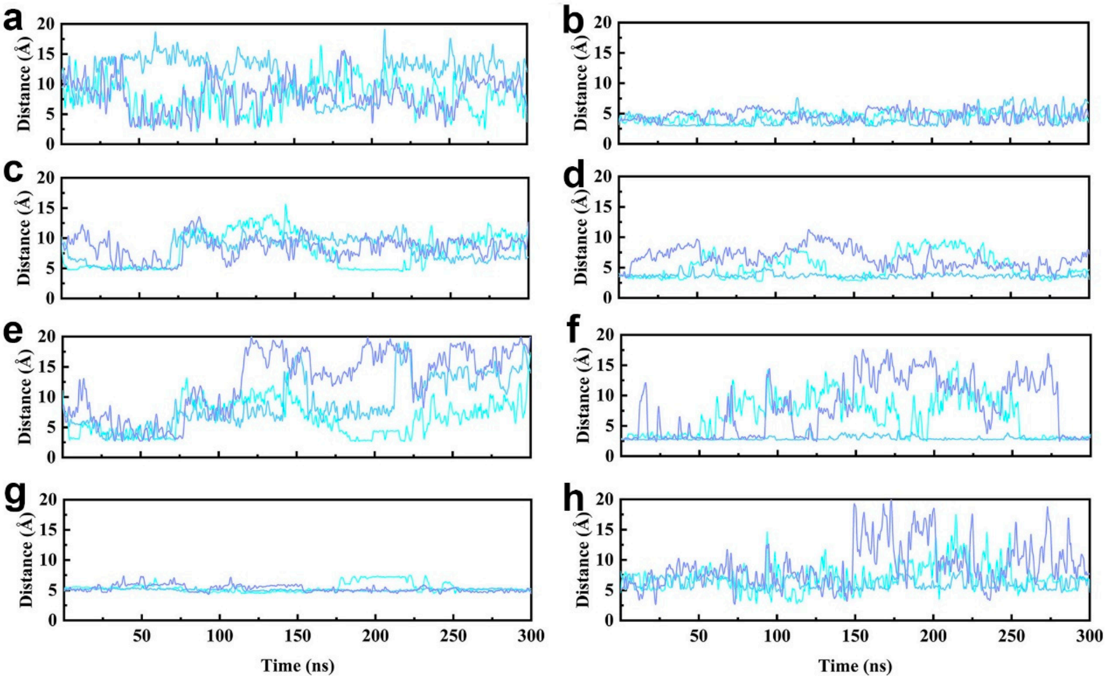

Figure S3. Distance evolution between the AST region and the kinase N-lobe in the inactive state. (**a**) Asp476 OD1–Lys194 NZ, (**b**) Glu481 OE2–Lys187 N, (**c**) Glu481 OE2–Lys206 NH2, (**d**) Glu481 O–Thr255 N, (**e**) Gln482 NE2–Val181 O, (**f**) Thr485 N–Glu254 OE2, (**g**) Lys487 NZ–Lys254 OE2, and (**h**) Lys487 NZ–Ser500 OG. The first, second, and third parallel trajectories of the inactive state are colored cyan, blue, and purple, respectively.

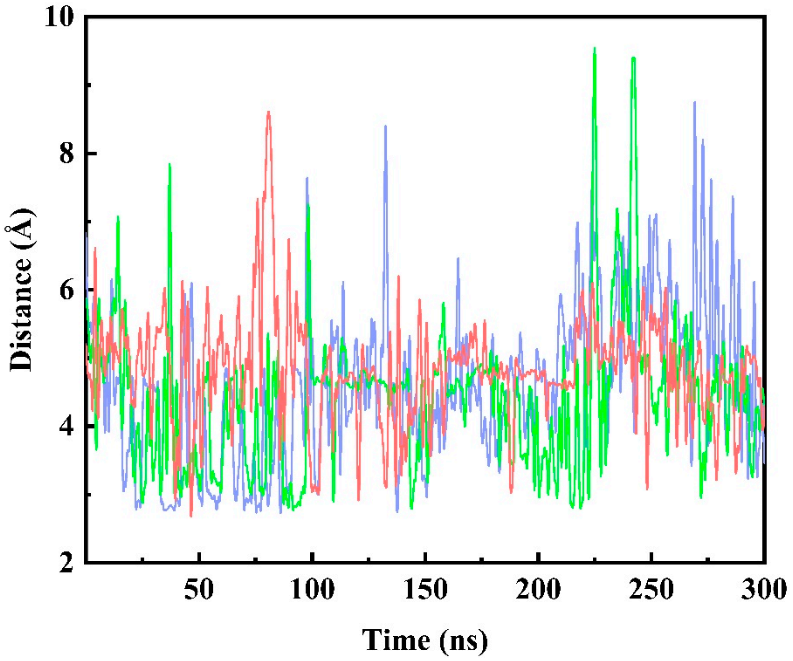

Figure S4. Distance evolution between Val486 and Lys220 in the active state, calculated by the closest non-hydrogen atoms. Specifically, the distance indicates Val486 O (AST) and Lys220 NZ (kinase domain N-lobe). The first, second, and third parallel trajectories of the inactive state are colored pink, red, and crimson, respectively.

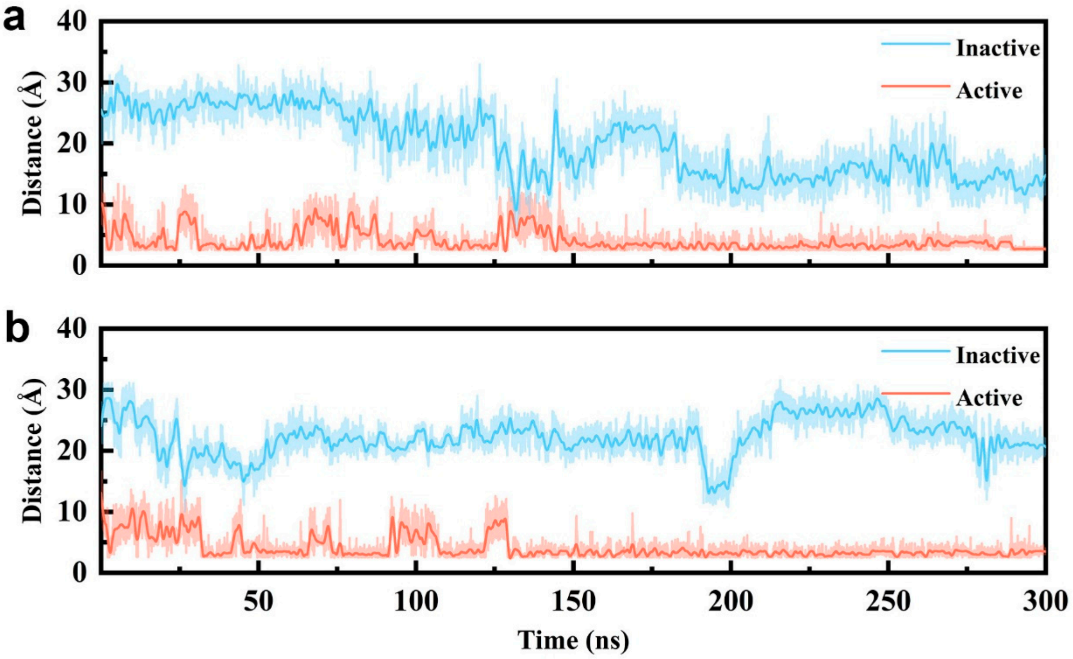

Figure S5. Distance change of Glu523–Arg23 for the second parallel 300-ns trajectory (a) and the third parallel 300-ns trajectory (b). The distance is calculated by the closest non-hydrogen atom, such as Glu523 OE2 atom and the Arg23 NH2 atom. The inactive and active states are colored blue and red, respectively.

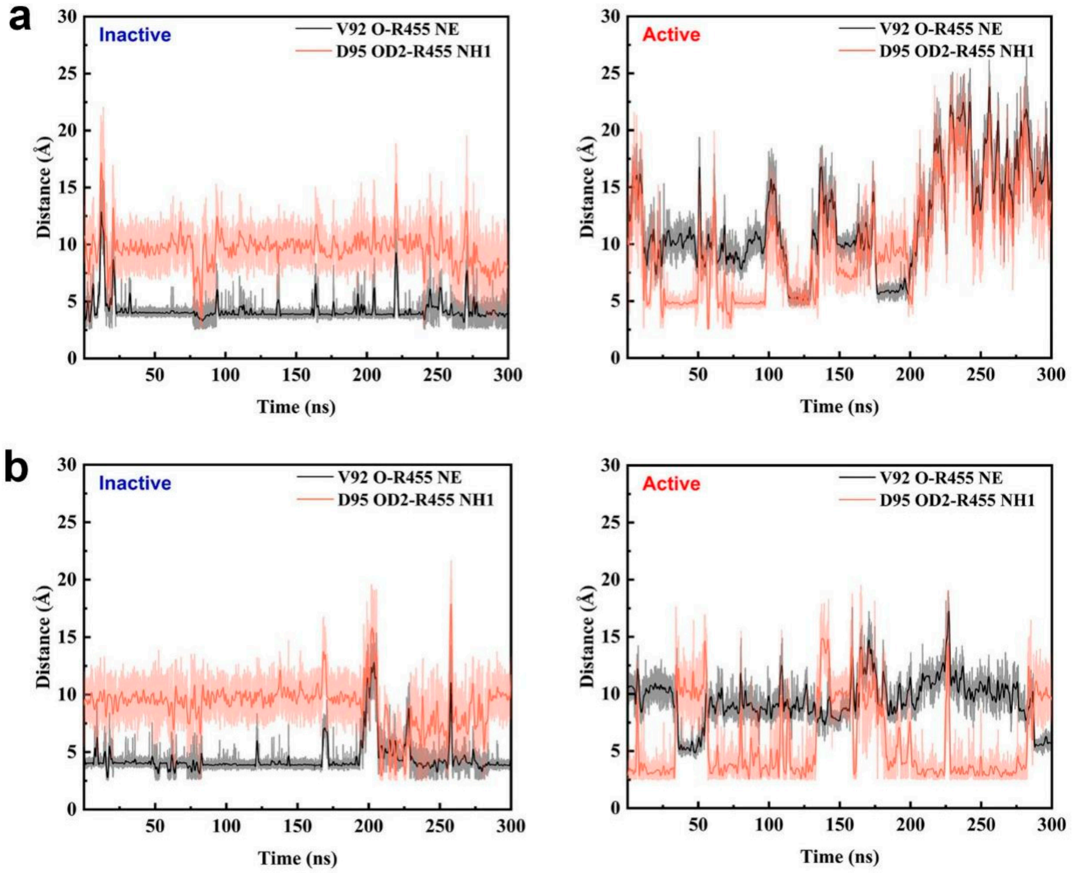

Figure S6. Activation-induced changes at the α5 helix and interface region residues in the additional parallel trajectories of GRK5. Distances between the Val92 O and Arg455 NE atoms (black), and the Asp95 OD2 and Arg455 NH1 atoms (red) for the second (a) and third (b) 300-ns parallel trajectories. In both panels, the left plot corresponds to inactive GRK5, and the right plot corresponds to active GRK5.
